# Ambulatory blood pressure measurement in the main cities of Cameroon: prevalence of masked and white coat hypertension, and influence of body mass index

**DOI:** 10.11604/pamj.2014.19.240.4887

**Published:** 2014-11-03

**Authors:** Noah Takah, Anastase Dzudie, Jules Ndjebet, Guela Wawo, Félicité Kamdem, Yves Monkam, Henry Luma, Kathleen Blackett Ngu, André Pascal Kengne

**Affiliations:** 1 Global Health Systems Solution, Limbe, Cameroon; 2 Department of Internal Medicine, Faculty of Health Sciences, University of Buea, Buea, Cameroon; 3 Department of Internal Medicine, Faculty of Health Sciences, University of Cape Town, South Africa; 4 Department of Internal Medicine, Douala General Hospital, Douala, Cameroon; 5 Douala cardiovascular centre of Bonapriso, Douala, Cameroon; 6 Faculty of Medicine and Biomedical Sciences, University of Yaounde 1, Yaounde, Cameroon; 7 Department of Internal Medicine, Yaoundé University Teaching Hospital Yaoundé, Cameroon; 8 South African Medical Research Council of South Africa, Cape Town South Africa Department of Medicine, University of Cape Town, Cape Town, South Africa; 9 Non-Communicable Diseases Research Unit, South African Medical Research Council, Cape Town, South Africa

**Keywords:** White Coat Hypertension, determinants, diagnosis, Ambulatory Blood Pressure Monitoring

## Abstract

**Introduction:**

Identifying White Coat Hypertension (WCH) may avoid inappropriate commitment of individuals to lifelong and costly blood pressure (BP) lowering medications’. We assessed the prevalence and determinants of WCH in urban clinical settings in Cameroon.

**Methods:**

Participants were a consecutive sample of adults, who underwent ambulatory BP measurements (ABPM) for the diagnosis of hypertension and evaluation of treatmentin three referral cardiac clinics in the cities of Yaounde and Douala, between January 2006 and July 2011. WCH was defined as an office-based systolic (or diastolic) BP ≥ 140(90) mmHg together with an average day time ambulatory systolic (and diastolic) BP < 135(85) mmHg.

**Results:**

Of the 500 participants included, 188 (37.6%) were women, 230 (46%) were nonsmokers and 53 (10.6%) had diabetes mellitus. The mean age was 51.6±10.2years. The ABPM readings were higher in men than in women (p<0.05).The prevalence of WCH was 26.4% overall, 39.3% in women and22.4% in men (p=0.01).In multivariable analysis, body mass index was the only significant determinant of WCH (Odds ratio= 1.15(95% confidence intervals: 1.00-1.43), p<0.05).

**Conclusion:**

The prevalence of WCH was high in our study population and was correlated only with BMI. Accurate measurement of BP and appropriate diagnosis of hypertension using ABPM in this setting may help limiting the consequences of over estimating hypertension severity on individuals, families and health systems.

## Introduction

Hypertension has been established as the commonest single risk factor for the occurrence of major cardiovascular events and deaths. It is a growing public health problem worldwide with a rapidly increasing prevalence in developing countries such as those in sub-Saharan Africa. The number of people affected is increasing so rapidly that some published data suggest that the prevalence of hypertension in developing countries is already matching that in developed countries [1–3]. Such a dramatic rise in the prevalence of hypertension would have as potential consequences an explosion of health care cost in sub-Saharan African countries that are already facing economic constraints [4–6]. Appropriate diagnosis of people with the disease is key to the success of hypertension management programs. However, accurate diagnosis of hypertension remains a real challenge especially when it is based on office measurements alone. Indeed, a substantial number of individuals, including those under treatment for hypertension, show a “white-coat” effect that could cause an overestimation of their real blood pressure (BP) levels [7]. Also, correlation between systolic (SBP) and diastolic (DBP) (BP) level and risk of target organ damage is stronger for ambulatory BP measurement (ABPM) than daytime office measurements and therefore ABPM has been recently recommended as the preferential tool to diagnose hypertension. The use of ABPM allows the identification of individuals with white coat hypertension and may help avoiding inappropriate commitment of individuals to lifelong and costly BP lowering medications [8].

The use of automated blood pressure measuring techniques has facilitated the accurate diagnosis of white coat hypertension. However, there are still very little information on the prevalence and correlates of WCH in Africa. This study was carried out to determine the prevalence of masked hypertension and WCH, and determinants of WCH in major urban clinical settings in Cameroon.

## Methods

This was a cross sectional hospital-based study carried out in three specialized cardiovascular clinics (Douala cardiovascular centre, Douala General Hospital and the cardiovascular centre at Hippodrome Yaounde) of the two major cities (Douala and Yaounde) of Cameroon. These towns were chosen because they represent the areas where most of internal medicine and cardiovascular specialists were located and were likely to request and interpret ABPM during the study period. The study was approved by the administrative authorities of the three health facilities, and ethical clearance was obtained from the Buea Faculty of Health Sciences Ethic Committee.

The study had two recruitment phases. First we retrospectively reviewed three hundred files of patients who had undergone ABPM from 1^st^ January 2009 to 31st December 2011. In the second recruitment phase (from 1^st^ January 2012 to 31^st^ July 2012), two hundred patients aged 18 years or above were referred to the recruiting centres for diagnosis of hypertension and/or evaluation of their antihypertensive medication. In the prospective phase, informed consent was obtained from the patients and those who accepted to undergo an ABPM and was included in the study.

For both recruitment phases the following variables were collected: sex, age, educational level, medical history hypertension, diabetes, and smoking with quantification of pack/years in smokers, height and weight for calculation of body mass index (BMI), office systolic and diastolic BP, drug history, mean day time systolic (diastolic) ambulatory BP, mean night time systolic (diastolic) ambulatory BP. Participants were stratified for alcohol consumptioninto occasional alcohol consumers, low, moderate and excessive alcohol consumers. Excessive alcohol consumption was based on intake of either more than 3 (2 for women) standard glasses of wine per day or more than 10 (5 for women) local beers (1 local beer contains 28 g of alcohol) per week. Moderate alcohol consumption was based on intake of 25-30 g per day. Those who smoked at least one cigarette per day at the time of consultation were classified as active smokers, and those who have smoked for at least 3 years in the past, but had stopped by the time of consultation, were classified as ex-smokers. The drug history included the number, name and class of the antihypertensive medications, and the time of the day the drug was taken.

The office BP measurement was taken by a trained nurse after 10 minutes of rest, using automated electronic sphygmomanometers (OMRON M3 HEM-7200-E Omron Matsusaka Co Ltd Kyoto Japan) with the patient in a seated position. BP was taken in both arms. Three consecutive BP measurements were taken and the average of the 3 values (using the higher pressure arm) was considered as the office BP. The 24h-ABPM was done using a commercially available system (Space Labs 90207 system). A typical weekday was chosen for different patients and normal daily activities were allowed. BP was recorded during the day for every 15minutes (from 07:00 to 21:00) and every 30minutes during the night (from 21:00 to 07:00). The mean systolic and diastolic BP was calculated for daytime and night time within 24hrs of recording. ABPM records were considered valid only if the number of BP recordings were at least 70% of the expected readings assessedas valid by the software analysis; otherwise patients underwent a second recording. Patients showing no valid readings for greater than two hours also underwent a second recording. Patients were classified as having WCH if their office systolic (diastolic) BP was ≥ 140 (90) mmHg and their mean daytime ambulatory systolic BP was < 135 mmHg and ambulatory diastolic BP was < 85 mmHg. Patients were classified as having masked hypertension if their systolic (diastolic) BP was ≤ 140 (90) mmHg and their mean daytime ambulatory systolic BP was > 135 mmHg and ambulatory diastolic BP was > 85 mmHg [9].

Data were analysed using the Statistical Package for Social Sciences (SSPS Inc, Chicago, Illinois, USA) version 17.0 software. Results are summarized as counts and percentages for qualitative variables and as mean and standard deviation (SD) for quantitative variables. Groups comparison used the Student t-test for quantitative variables and the chi-square test for qualitative variables. To assess the determinants of WCH, multivariate logistic regression models were used. A p-value

## Results

**Figure 1:**
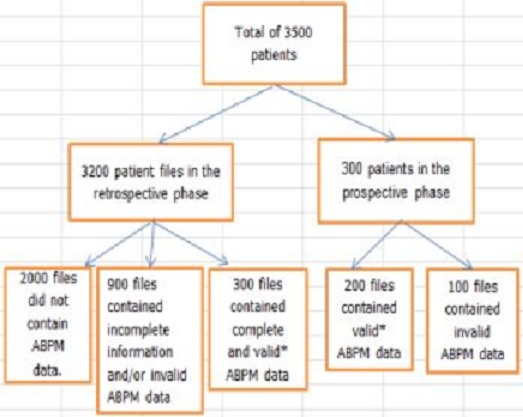
Flow chart showing adoption of ABPM data

A flow chart of adoption of ABPM data is shown in Figure 1. A total of 500 patients were included among whom 312 (62.4%) were females, 230 (46%) were non-smokers, 199 (39.8%) had a positive family history of hypertension, and 53(10.6%) had type 2 diabetes. The mean age was 51.6±10.2 years, the mean BMI was 29.3±4.2 kg/m^2^ (Table 1). As expected, mean office-basedBP was high (systolic 154.5±21.8 vs. diastolic 92.7±13.1 mm Hg). The mean daytime ambulatory systolic blood pressure was 140.9±16.4 mmHg and the mean daytime ambulatory diastolic blood pressure was 89.0±10.8 mmHg (Table 1). The clinic blood pressures were similar for males and females. Day and night time ambulatory blood pressure readings were lower for females (p<0.05) (Table 1).

**Table 1:** General and blood pressure characteristics of our study population

Variable	All patients(n = 500)	Males (n = 312(62.4%))	Females (n = 188(37.6%))	P value
Age[mean(SD) in years]	51.6(10.2)	51.8(8.8)	51.6(11.7)	0.7
Body mass index [mean(SD)in kg/m2]	29(4.2)	28.9(3.8)	29.9(4.6)	0.01
Diabetes (%)	10.6	13.1	6.4	0.01
Family history of HTN (%)	39.8	34.9	47.9	0.01
Clinic BP[Mean(SD)]				
Systolic	154.5(21.8)	154.4(19.7)	154.5(25)	0.9
Diastolic	92.7(13.1)	92.7(12.5)	92.7(14.0)	0.9
24H Ambulatory BP[Mean(SD)]				
Systolic	138.8(16)	140(15.7)	136.7(16.4)	0.02
Diastolic	86.7(10.8)	87.7(10.1)	85.1(11.7)	0.09
Daytime Ambulatory BP[Mean(SD) mmHg]				
Systolic	140.9(16.4)	142.3(16.0)	136(16.4)	0.006
Diastolic	89.0(10.8)	91.2(11.2)	86(11.0)	0.004
Night time Ambulatory BP[Mean(SD) mmHg]				
Systolic	133.1(17.4)	135.2(17.1)	130(17.0)	0.07
Diastolic	80.8(11.8)	84.6(11.0)	82(11.4)	0.03

**Figure 2:**
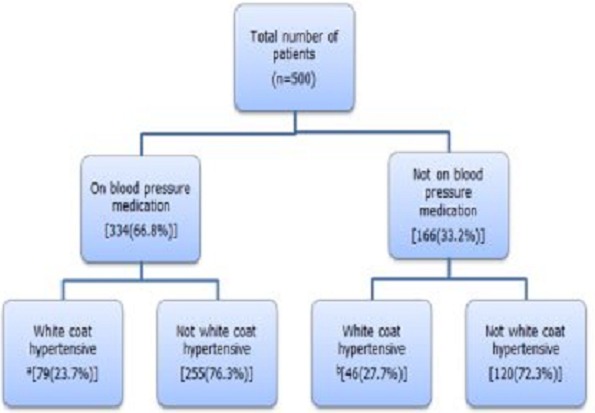
Flow diagram showing the percentage of white coat hypertensive individuals and their treatment status

The distribution of various sub forms of hypertension was: masked hypertension (MHT):9%, normotension (NT):18%, WCH: 25% and sustained hypertension (SHT): 49%. Of the 125 patients presenting with WCH, 79 (23.7%) were on BP lowering medication (Figure 2).

**Figure 3:**
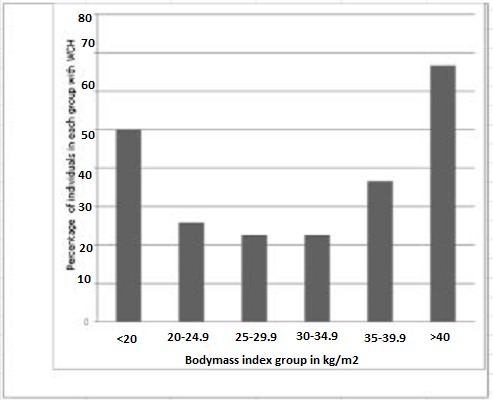
Bar chart showing the Percentages of individuals with white coat hypertension in different body mass index groups

In both univariable and multivariable adjusted logistic regression models, the main driver of white coat hypertension was BMI (Table 2). The prevalence trend of WCH showed a U-shape with a higher prevalence in groups of BMI lower than 20 or greater than 40 kg/m2 (Figure 3). Furthermore, a kg/m2 higher BMI was associated with an odd ratio (95% confidence interval) of 1.15 (1.00-1.43) for prevalent WCH. WCH was not significantly associated with age, sex, diabetes, smoking status, alcohol consumption or family history.

**Table 2:** Unadjusted and adjusted odd ratio and 95% confidence intervals for predictors of White Coat Hypertension in our study population

Variables	WCH Unadjusted	WCH Adjusted for age and sex
Age group (Reference group < 35 years)	0.88 (0.55-2.15)*	
Sex (men vs. women)	1.16 (0.69-1.96)	
Smoking	0.89 (0.52-1.53)	0.89 (0.48-1.66)
Family history of hypertension	1.02 (0.34-1.38)	0.76 (0.33-1.73)
Alcohol consumption		
Low	3.12 (0.34-31.38)	3.96 (0.91-34.73)
moderate	1.60 (0.14-11.38)	1.44 (0.16-12.94)
Excessive	1.12 (0.26-18.38)	1.94 (0.28-17.34)
Diabetes	2.19 (0.95-4.38)	2.55 (0.99-5.55)
BMI	1.10 (1.01-1.41)*	1.15 (1.00-1.43)*
Office SBP	1.10 (0.95-1.28)	1.00 (0.99-1.02)
Office DBP	1.08 (0.88-1.13)	0.98 (0.96-1.06)

* p<0.05; BMI, Body Mass Index. High BMI was positively correlated with having WCH

## Discussion

In this cross sectional study of hypertensive groups of patients from Douala and Yaounde,Cameroon,we found a high prevalence of WCH. This high prevalence was largely influenced by adiposity. To our knowledge, this is the largest study of ABPM in a sub-Saharan African setting and the used methodology provides findings that are comparable to other studies onWCH. Our findings provide some suggestions on the segment of the population to be targetted by further investigations via ABPM for the purpose of diagnosing and treating hypertension in our resources-limited settings.

The level of ABPM systolic BP(SBP) and diastolic BP(DBP) were high compared to other studies such as the PAMELA study and Australian data. The mean clinic BP in our study was 27/11 mmHg and 13/11 mmHg higher than in the PAMELA and Australian study respectively. Furthermore,the mean 24 H ABP readings in our study was 21/13 mmHg and 7/5 mmHg higher than in the PAMELA and Australian studies respectively [10, 11]. This observation may reflect certain peculiarities in our study population that require further investigation in future studies with larger databases.

The overall prevalence of WCH in our study population was 25.0%. This was higher than the 15.4% reported by Dolanand associates [12] in Europe as well as Godil and associates [13] who reported a prevalence of 16.6% in Asia (Pakistan);but lower that the 33% reported byAbir-Khalil and colleagues in North Africa (Morocco). Differences in terms of definition of WCH and cut- off points used may account for these differences in prevalence. Verdecchia and colleagues in 1992 reported that depending also on the selection of patients and groups of patients, the prevalence of white coat hypertension can vary from 18% to 60%. The prevalence of White coat hypertension seems to lower in those with chronic kidney disease [14, 15]. In a case where no distinction is made between WCE and WCH, the prevalence of WCH can be higher than the onefound in this study. This high prevalence of WCH was largely influenced by some risk factors that deserve attention. Suspected WCH is an established indication for ambulatory blood pressure monitoring. Understanding the likely profile of individuals with WCH will facilitate the selection of patients for ABPM. Some studies have been carried out elsewhere to assess the predictors (determinants)of WCH. Among these studies, Honda and colleagues 2003 [14], assessed determinants of white coat hypertension such as age, gender, BMI. We expanded this panel of candidate predictors of WCH to include smoking, family history of hypertension, level of alcohol consumption and clinic blood pressure could also be determinants.

Studies of the association between BMI and ABPM variables have shown conflictingfindings. In a large retrospective study of 3928 patients referred for ABPM, Ben-Dov et al [16] found no association between BMI and WCH. Other reports support the findings of Ben-Dov et al [17, 18]. When our study population was categorized according to their BMI, the prevalence trend showed a U shape with a higher prevalence at both extremes of the BMI distribution and BMI was the only determinant of WCH in this population. The increasing prevalence of WCH with increasing BMI is similar to other reports of BMI as a determinant of WCH and/or WCE (white coat effect) [14, 19]. Differences in population study and WCH as well as WCE definitions might account at least partially for these contradicting results. Another explanation is that obese patients, who usually have higher office BP, would be expected toexhibit larger clinic-awake differences, unless the data are adjusted for office BP. In our study, the influence of BMI on WCH was independent of the systolic (diastolic) BP. This is likely to be a characteristic of our population, which needs confirmation in a larger study especially for the lower BMI group.

### Strengths and limitations of the study

The strengths of our study include the multicentre nature. It was carried in key centres highly engaged in performing ABPM, the rigorous data collection; the sample size was good and comparable to many other large database studies elsewhere. However, participating centres were based only in urban areas, and whether findings from this study apply to people in rural settings is unknown. Furthermore, the non-random selection of participants in the study suggests that our estimates of WCH could possibly carry some bias. Such a bias however, if any, is unlikely to affect the association of WCH with adiposity and may therefore not alter the main conclusion from this study.

Despite theses hort comings, we do believe the present study has relevance both for clinical and public health reasons. Given the high prevalence of hypertension in the Cameroon population as largely reported in previous studies, the findings of our study are indicative of the important role of an accurate measurement of BP and diagnosis of hypertension using either ABPM or HBPM so as to reduce unnecessary commitment to a lifelong treatment with BP lowering medications. That up to a quarter of individuals labelled as hypertensive in this setting actually has WCH suggest by the way of simple arithmetic that over a quarter of resources dedicated to hypertension care in this setting are resources inappropriately used. Interestingly however, our study also suggest that this waste of resources could potentially be reduced just by considering for further investigation via ABPM, those in the upper tail of the BMI distribution who are diagnosed with hypertension based on office measurements.

## Conclusion

In conclusion, our study has provided findings showing a high prevalence of white coat hypertension in Cameroon which appear to be driven essentially by adiposity. A policy in this setting favouring the consideration of people with elevated BMI diagnosed with hypertension using office measurements, for further investigation via ABPM has a potential to limit the number of those who may unnecessarily be committed to chronic medications to control the BP levels.
